# Excess fermentation and lactic acidosis as detrimental functions of the gut microbes in treatment-naive TB patients

**DOI:** 10.3389/fcimb.2024.1331521

**Published:** 2024-02-19

**Authors:** Milyausha Yunusbaeva, Liliya Borodina, Darya Terentyeva, Anna Bogdanova, Aigul Zakirova, Shamil Bulatov, Radick Altinbaev, Fanil Bilalov, Bayazit Yunusbayev

**Affiliations:** ^1^ Laboratory of Evolutionary Biomedicine, International Institute “Solution Chemistry of Advanced Materials and Technologies”, ITMO University, Saint Petersburg, Russia; ^2^ Institute of Translational Biomedicine, Saint Petersburg State University, Saint Petersburg, Russia; ^3^ Department of Tuberculosis Monitoring, Republican Clinical Antituberculous Dispensary, Ufa, Russia; ^4^ Laboratory of Molecular Epidemiology and Evolutionary Genetics, Saint Petersburg Pasteur Institute, Saint Petersburg, Russia; ^5^ Laboratory of Neurophysiology of Learning, Institute of Higher Nervous Activity and Neurophysiology, Russian Academy of Sciences, Moscow, Russia; ^6^ Laboratory of Molecular Genetics, Republic Medical Genetic Centre, Ufa, Russia; ^7^ Department of Public Health and Health Organization with a course of ICPE, Bashkir State Medical University, Ufa, Russia

**Keywords:** tuberculosis, gut microbiome, dysbiosis, anaerobic fermentation, gut-derived lactic acidosis

## Abstract

**Introduction:**

The link between gut microbiota and host immunity motivated numerous studies of the gut microbiome in tuberculosis (TB) patients. However, these studies did not explore the metabolic capacity of the gut community, which is a key axis of impact on the host’s immunity.

**Methods:**

We used deep sequencing of fecal samples from 23 treatment-naive TB patients and 48 healthy donors to reconstruct the gut microbiome’s metabolic capacity and strain/species-level content.

**Results:**

We show that the systematic depletion of the commensal flora of the large intestine, *Bacteroidetes*, and an increase in *Actinobacteria, Firmicutes*, and *Proteobacteria* such as *Streptococcaceae, Erysipelotrichaceae, Lachnospiraceae*, and *Enterobacteriaceae* explains the strong taxonomic divergence of the gut community in TB patients. The cumulative expansion of diverse disease-associated pathobionts in patients reached 1/4 of the total gut microbiota, suggesting a heavy toll on host immunity along with MTB infection. Reconstruction of metabolic pathways showed that the microbial community in patients shifted toward rapid growth using glycolysis and excess fermentation to produce acetate and lactate. Higher glucose availability in the intestine likely drives fermentation to lactate and growth, causing acidosis and endotoxemia.

**Discussion:**

Excessive fermentation and lactic acidosis likely characterize TB patients’ disturbed gut microbiomes. Since lactic acidosis strongly suppresses the normal gut flora, directly interferes with macrophage function, and is linked to mortality in TB patients, our findings highlight gut lactate acidosis as a novel research focus. If confirmed, gut acidosis may be a novel potential host-directed treatment target to augment traditional TB treatment.

## Introduction

Tuberculosis (TB), caused by *Mycobacterium tuberculosis*, is one of the leading infectious disease killers worldwide. In 2021, according to the World Health Organization (WHO), 10.6 million people were diagnosed with tuberculosis, an increase of 4.5% from 2020 (World Health Organization 2021) Host immunity plays a central role in combating *M. tuberculosis* (MBT). Despite progress in elucidating the key elements of the innate (Liu et al., 2017) and adaptive immune responses to TB infection (Dheda et al., 2010) (Scriba et al., 2017), host factors governing susceptibility to infection are poorly understood. It is unclear why some individuals in TB-endemic countries never become infected with MBT or why most latently infected individuals never progress to active TB disease. Among factors contributing to host susceptibility, the intestinal microbiota is gaining attention due to its prominent role in modulating host immunity. There is growing evidence that the gut microbiota plays a key role in developing the host immune system (Hooper et al., 2012; Liu et al., 2017) In connection to tuberculosis, several recent studies have attempted to assess the potential role that the gut microbiota can play in the host’s susceptibility to TB, both in humans and in model organisms (Cardona et al., 2015; Majlessi et al., 2017). Specifically, experiments using model organisms suggest that gut microbiota can modulate host immunity and that microbiome changes can impact TB’s outcome and prognosis (Round and Mazmanian, 2010; Winglee et al., 2014; Negi et al., 2020). Despite these insights from model organisms, our understanding of the gut community in TB patients is limited to descriptive data on taxonomic alterations. Crucially, the functional impact of the microbiota on host immunity, mediated through microbial metabolic output, is lacking. Thus, based on 16S rRNA sequencing, earlier studies reported gut dysbiosis and discussed the possible impact of the detected taxonomic changes on the host (Hu et al., 2019; Shi et al., 2021; S. Wang et al., 2022; Yu et al., 2023). Such taxonomic descriptions are not enough to understand microbial function. There is increasing appreciation that gut microbes impact the human host more frequently via metabolic products (Rooks and Garrett, 2016). Therefore, insight into the metabolic capacity of the gut community is important to evaluate the potential effect of the microbiome on host immunity.

In this study, we used deep shotgun sequencing of fecal microbiomes in 23 treatment-naive TB patients and 48 healthy donors to dissect not only “who is present” in the gut community at the species/strain level but also infer “what they can do” by constructing metabolic functions from microbial genes. We showed that strong alterations of the gut communities in TB patients are accompanied by an increased amount of metabolic pathways indicative of microbial cell division and growth. A putative increase in bacterial turnover in patients is likely accompanied by an increased capacity for glycolysis and fermentation of glucose to produce acetate and lactate. Notably, pathways for lactic acid production were among the top changes in TB patients’ gut microbiome. Published evidence suggests that elevated serum lactate correlates with lung pathology and higher mortality (Ntambwe and Maryet, 2012; Im et al., 2021). Recent findings explain this detrimental effect by direct interference of lactic acid with macrophage activity against MTB and increased tissue destruction that worsens pathology. Thus, given our findings, more research is needed to understand whether the increased gut microbial potential for anaerobic glycolysis and fermentation has a link to elevated serum lactate in TB patients reported in the literature.

## Methods

### Study design and patient information

Our work is a case-control study with 23 treatment-naive TB patients and 48 healthy controls (HCs). For our dataset, we estimated the effect size (Cohen’s d) of the tuberculosis status on key microbiome parameters that can be directly compared to other studies. Based on the estimated effect size (Cohen’s d), we then performed a *post-hoc* power analysis for our sample size using the Evident software (Rahman et al., 2022). TB patients were recruited at the Republican Clinical TB Dispensary (Ufa), Russia, from 2019 to 2021. All patients had newly diagnosed active pulmonary TB as confirmed by chest radiography and a positive sputum smear or were positive for *M. tuberculosis* based on the GeneXpert MTB/RIF test without evidence of rifampin resistance. Exclusion criteria included serious comorbidities, hemoptysis, hypoxia, extrapulmonary tuberculosis, history of tuberculosis treatment, use of anti-TB drugs within the past 30 days, pregnancy or breastfeeding, and HIV infection.

Participants for the control group (n = 48) were recruited from staff working at the Institute of Biochemistry and Genetics of the Russian Academy of Science (Ufa, Russia), considering the matched age and sex of the enrolled TB cases. These control subjects had no unexplained symptoms or other medical conditions, such as infectious, autoimmune, or gastrointestinal diseases. All subjects had no change in chest radiography.

We collected freshly frozen samples from 48 healthy adult donors and 23 treatment-naive TB patients. Fecal samples from TB patients were taken before treatment started. All participants signed an informed consent form before entering the study.

### Fecal sample collection and DNA extraction

Fresh stool samples from the participants were collected using sterile containment and stored at 80°C immediately for further analysis. Metagenomic DNA was extracted from 200 mg of feces using the QIAamp DNA Stool Mini Kit (QIAgen, Netherlands).

### Metagenomic sequencing

Sequencing libraries were generated using the TruSeq DNA PCR-Free Sample Preparation Kit (Illumina, USA), following the manufacturer’s recommendations. The sequencing libraries were then sent for sequencing using the Illumina HiSeq2500 instrument, and at least 15 million 150 bp paired-end reads were generated for each donor.

### Raw sequence QC and preprocessing

We examined sequence quality before and after quality trimming and decontamination using FastQC v0.11.9 (Wingett and Andrews, 2018). Trimmomatic version 0.33 (Bolger et al., 2014) was used to remove low-quality bases (Q20), and fastp v0.23.2 was used to clip Illumina adapters (Chen et al., 2018). We additionally removed tandem repeats using Tandem Repeats Finder Version 4.09 (Benson, 1999). Finally, the KneadData v0.10.0 pipeline of the bioBakery toolset was used to decontaminate reads originating from the human genome, transcriptome, and microbial RNA (McIver et al., 2018). Specifically, we used the human reference genome (build hg37), the human reference transcriptome (build hg38), and the SILVA ribosomal RNA reference databases.

### Bacterial community diversity and principal component analysis

Shannon alpha and beta diversity measures were estimated using the microbiome R package (Lahti and Shetty, 2017). Differences in Shannon alpha diversity between patients and controls were tested using the two-sample Kolmogorov-Smirnov test implemented in the ks.test() function of the stats R package (R Core Team, 2022). To visualize gut community differences between donors using major axes of microbial variation, we applied principal component analysis (PCA) on the species abundance matrix. To adapt sparse data on relative abundance for principal component analysis, we imputed zeros in the relative abundance table using the cmultRepl() function in the zCompositions R package version 1.4.0-1 (Palarea-Albaladejo and Martín-Fernández, 2015). Next, to take compositionality into account, the relative abundance table was transformed using the center log ratio (CLR) prior to PC analysis. PC analysis was carried out using the prcomp() function in the stats R package (R Core Team, 2022).

### Inference of taxonomic content of the gut microbiome and between-group comparisons

Prior to downstream analyses, all bacterial taxa observed only in a single donor and at a relative abundance of less than 0.0001 were discarded. The choice of an abundance threshold ensured that we kept rare bacterial species that can impact human disease, as demonstrated in recent work (Chriswell et al., 2022). Metaphlan v4.0 tool was used to infer both known bacterial species and strains as well as uncharacterized bacterial species. Uncharacterized bacterial species in the metaphlan v4.0 tool are defined in terms of species-level genome bins (SGBs) using so-called metagenomically assembled genomes (MAGs) (Blanco-Míguez et al., 2023). To facilitate comparison with published data, we analyzed relative abundances of bacterial taxa in sampled groups (TB patients and controls) at different taxonomic levels by aggregating strains and species into genus, family, order, class, and phylum. We used linear discriminatory analysis with effect size estimation (LEfSe) implemented in the microbial R package to identify bacterial taxa that characterize differences between patients and controls. We also computed the average proportion of each taxon between patients and controls at different levels (e.g., phylum and class). A statistical difference in proportions between groups was tested using the ANCOM statistical framework (Mandal et al., 2015) using the FDR (false discovery rate) approach to adjust p-values. When we tested average proportions using ANCOM, we were interested in comparing dominant taxa between groups. Therefore, we required bacterial taxa to be present in 25% of donors at an abundance of 5% or more.

### Inference of the microbial metabolic capacity of the gut microbiome and between-group comparisons

The metabolic capacity of the microbiome, often referred to as the metabolic profile (potential), was inferred using the HUMAnN 3.6 framework using default settings. Briefly, HUMAnN 3.6 compares the sequences from donor metagenomes to known microbial genomes and genes to infer known metabolic reactions and aggregate them into known microbial pathways. Specifically, for bioinformatic queries, HUMAnN 3.6 uses the ChocoPhlAn 3 database of 99.2 thousand annotated reference genomes from 16.8 thousand microbial species in the UniProt database (January 2019) (UniProt Consortium, 2019) and the corresponding functionally annotated 87.3 million UniRef90 gene families (Suzek et al., 2015). For each input donor (fecal) metagenome, HUMAnN 3.6 provides bacterial-specific genes and pathway abundance whenever genes can be attributed to known bacterial species in the database. Gene families are annotated by default using UniRef90 definitions and pathways using MetaCyc definitions (Caspi et al., 2020). Whenever inferred genes cannot be attributed to bacterial species, gene and pathway abundances are reported without specifying their bacterial provenance. To identify metabolic pathways that are differentially abundant in patients or controls, we used linear discriminatory analysis with effect size estimation (LEfSe) implemented in the microbial R package. For pathways that showed strong enrichment in patients (pathways with the largest effect size, the LDA score), we also inferred bacterial species, genera, and higher-level taxa that were major contributors to that pathway.

## Results

### Baseline characteristics

A total of 23 untreated TB patients and 47 healthy controls were recruited in this study (**Supplementary Table 1**). There was no significant difference in sex or age between the participating TB patients and HCs (p < 0.05, **Supplementary Table 1**). The median age of the analyzed patients was 43 (IQR 34.5–55), and male TB patients significantly outnumbered females (61% vs. 39%). The patients were diagnosed with infiltrative tuberculosis (65.2%), disseminated tuberculosis (30.4%), and caseous pneumonia (4.3%).

### The gut microbiome composition in TB patients is strongly divergent from that in healthy controls

**Figure 1:**
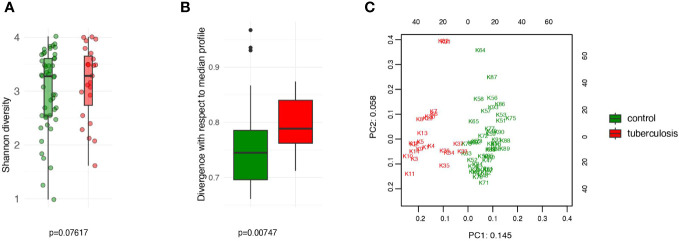
Comparison of the intestinal microbiota diversity in untreated TB patients versus healthy controls. **(A)** The alpha diversity was assessed using the Shannon index. No significant differences in diversity between the TB and control groups (P > 0.05); **(B)** The beta diversity, assessed as Bray-Curtis dissimilarity, indicates significant divergence between the TB patients and controls (P < 0.007); **(C)** PCA analysis based on CLR-transformed relative abundance. PC1 and PC2 are given with the proportion of total variance explained.

To obtain a comprehensive description of the gut microbiome, we searched for known (isolated) and unknown microbial taxa inferred from metagenome-assembled genomes (MAGs). Unlike traditional approaches that provide a truncated picture of the microbial diversity, this novel approach, implemented in Metaphlan 4.0 (Blanco-Míguez et al., 2023), provides a more complete description of the microbial content of the gut. We detected 987 microbial taxa in our combined set of treatment-naive TB patients and HCs. Our data show that gut community diversity (alpha-diversity) is not reduced in treatment-naive TB patients (**Figure 1A**) but shows statistically significant divergence (Bray-Curtis dissimilarity) from the gut community in HCs (p = 0.00747) (**Figure 1B**). We next showed that the extent of community divergence is strong enough to separate patients from controls in an unsupervised manner using principal component analysis (PCA). The principal component analysis clearly separated TB patients from HCs along the first PC (**Figure 1C**). Clear separation along the first PCs, the strongest axis of microbial variation, suggested that tuberculosis has a large effect size. To compare with common factors and diseases that impact gut microbiome, we estimated standardized effect size in terms of Cohen’s d. For TB status, effect size on PCs based on taxonomic and metabolic pathways varied between 4.5 and 1.8, respectively. TB, therefore, impacts the gut microbiome much stronger than autoimmune diseases (Cohen’s d=0.2), and many known confounding factors, such as diet and lifestyle factors, reported in the American Gut Project (Cohen’s d did not exceed 0.2 and Cohen’s f <0.1, Note that f=2d) (McDonald et al., 2018; Rahman et al., 2022). Our estimates show that TB status has a relatively large effect size on microbiome composition, and our moderate sample set provides acceptable power in this scenario of large effect size (**Supplementary Figure 1A**). Our results suggest that untreated TB patients arrive at the hospital with a strongly disturbed gut community.

### Differentially abundant bacterial taxa between TB patients and healthy controls

**Figure 2:**
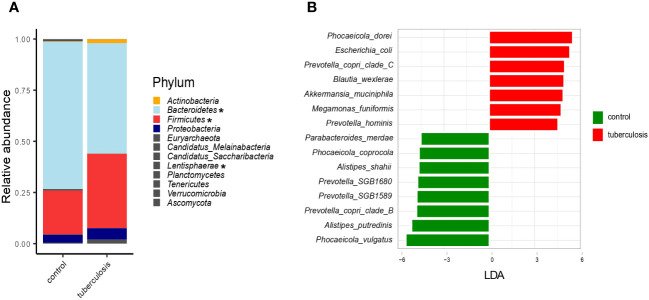
Taxonomic composition of the fecal microbiota of TB patients and healthy controls. **(A)**. The average relative abundance of bacterial taxa at the phylum level in TB patients and HCs. Asterisks indicate taxa that showed statistically significant differences between the TB group and HCs based on the ANCOM statistical analysis (P < 0.05); **(B)**. Barplot depicting top bacterial species (LDA scores > 4.5) discriminating between groups.

We characterized microbiomes at different taxonomic resolutions to facilitate comparison with published data. At the phylum level, TB patients showed an apparent increase in *Firmicutes* abundance (36.4% vs. 21.3% in controls, p = 0.03) and an elevated proportion of *Actinobacteria* (1.8% vs. 0.2%, p = 0.0002) and *Proteobacteria* (5.6% vs. 3.8%, p > 0.05) (**Figure 2A**). This increase in *Firmicutes* was at the expense of *Bacteroidetes* (54% vs. 72.2%), a frequent sign of a disturbed gut microbiome. Taxonomic composition at the genus, family, and order levels was also strongly altered in TB patients (**Supplementary Figure 2**).

We next used LEfSe, a linear discriminant analysis, to identify the combination of microbial species that differentiate TB patients’ gut microbiomes from HCs (Segata et al., 2011). For each taxon, LEfSe also estimates the effect size, the LDA-score. Altogether, 205 taxa (species and subclades defined by MAGs) showed differential abundance (adjusted p-value ≤ 0.05 and LDA-score ≥ 2.5) (**Supplementary Table 2**, **Supplementary Figure 3**), and we highlighted bacterial species with the strongest differentiation (LDA ≥ 4.5) (**Figure 2B**). For example, *Phocaeicola dorei, Escherichia coli, Prevotella copri clade C*, and *Akkermansia muciniphila* were strongly enriched in TB patients (**Figure 2B**). In contrast, *Phocaeicola vulgatus, Alistipes putredinis, Prevotella copri clade B*, and *Prevotella SGB1589* were more abundant in the HCs (**Figure 2B**). The top TB-associated taxon, *Phocaeicola (Bacteroides) dorei*, was previously isolated from patients with bacteremia (Cobo et al., 2022). Despite wide interest in the role of *P. (B.) dorei* in the intestinal microbiome, this bacterium is poorly studied, and there is little evidence of its direct involvement in the pathogenesis of infections (Yoshida et al., 2018; Cobo et al., 2022). Its accurate identification is challenging due to its close relationship with *P. vulgatus* (96% similarity), one of the most numerically predominant *Bacteroides* species in the human intestine (Pedersen et al., 2013; Vu et al., 2022). Unlike *P. (B.) dorei*, other top TB-associated species, such as *E. coli, A. muciniphila*, and *M. funiformis*, are relatively better studied and were previously implicated in various diseases, such as inflammatory bowel diseases (Crohn’s disease and colitis), metabolic syndromes (obesity, hypertension, type 2 diabetes), autism, and infections (diarrhea, cystitis) (Sheng et al., 2022) (Zou et al., 2020).

**Figure 3:**
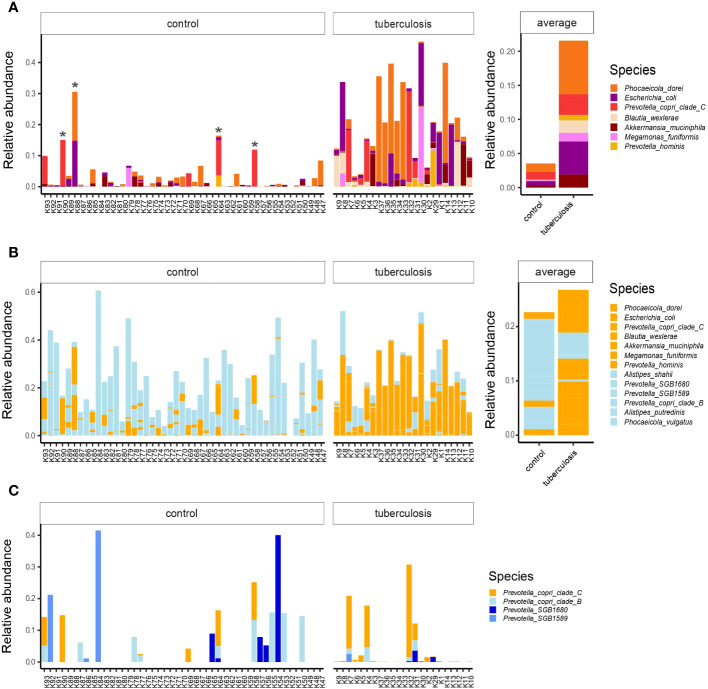
The relative abundances of top TB-associated species in the gut microbiota of TB patients and healthy controls. **(A)** The relative abundances of top TB-associated species (LDA scores > 4.5) in TB patients and HCs lined up on the x-axis with respective IDs. Asterisks indicate outlier healthy donors carrying a high proportion of TB-associated species; Averaged abundances are shown on the right-hand side barplot; **(B)** Combined relative abundances of TB-associated species (potential pathobionts) and health-associated commensals in donors. In orange potential pathobionts, in light-blue - health-associated commensals; **(C)** The distribution of *Prevotella copri* clades and related lineages in the gut microbiota of TB patients and healthy controls. *Prevotella copri clade C* in orange was associated with TB, while clades in blue were more prevalent in healthy donors.

We next inspected the relative abundance of these top discriminatory species. We noted that some healthy donors carried high proportions of TB-associated species, such as *Phocaeicola dorei, Escherichia coli*, and *Prevotella copri clade C* (**Figure 3A**, asterisk). Our bacterial culturing test for these healthy donors showed overgrowth of *C. albicans* or lactose-negative *E. coli*, and the coprogram stool test showed iodophilic flora, a sign of an imbalance between normal and pathogenic gut flora (our unpublished data). This additional insight into healthy outliers’ microbiomes, together with published data on some of the discriminatory species (Zou et al., 2020; Sheng et al., 2022), suggest that the top TB-associated species are pathobionts that correlate with intestinal dysbiosis and poor health. Notably, these potential pathobionts comprise a quarter of the gut microbiome in some TB patients (shown in orange in **Figure 3B**), a high toll on the host immunity given the strong deficit in health-associated commensals.

### Depleted strain diversity of common gut commensals in TB patients

We used strain-level profiling with Metaphlan 4 (Blanco-Míguez et al., 2023) and uncovered subspecies diversity that showed contrasting enrichments in TB patients and HCs. For example, according to linear discriminant analysis, *Prevotella copri clade C* proportion was higher in TB patients, while *Prevotella copri clade B* was associated with controls (**Figure 2B**; **Supplementary Table 2**). Closer inspection revealed that healthy donors featured a more diverse collection of *Prevotella* subclades and lineages than TB patients (**Figure 3C**). We looked for published data to see if *Prevotella copri* clades are associated with human diseases. While earlier studies reported conflicting correlations between *Prevotella copri* and various diseases (Dillon et al., 2014) (Pedersen et al., 2016), a recent large-scale study did not support these earlier claims (Tett et al., 2019). Instead, Tett and colleagues found that *Prevotella copri* clade diversity was reduced in much of the world’s populations with Westernized diets and disease settings, while non-industrialized societies with fiber-rich traditional diets featured richer diversity (Tett et al., 2019). Our study shows that TB patients show reduced diversity of *Prevotella copri* clades. We, therefore, hypothesize that *Prevotella copri clade C’s* association with TB is likely driven by a depletion of other lineages (**Figure 3C**) but not by a detrimental role of this particular clade.

### The metabolic potential of the gut microbiota in TB patients points to increased fermentation and lactic acidosis

**Figure 4:**
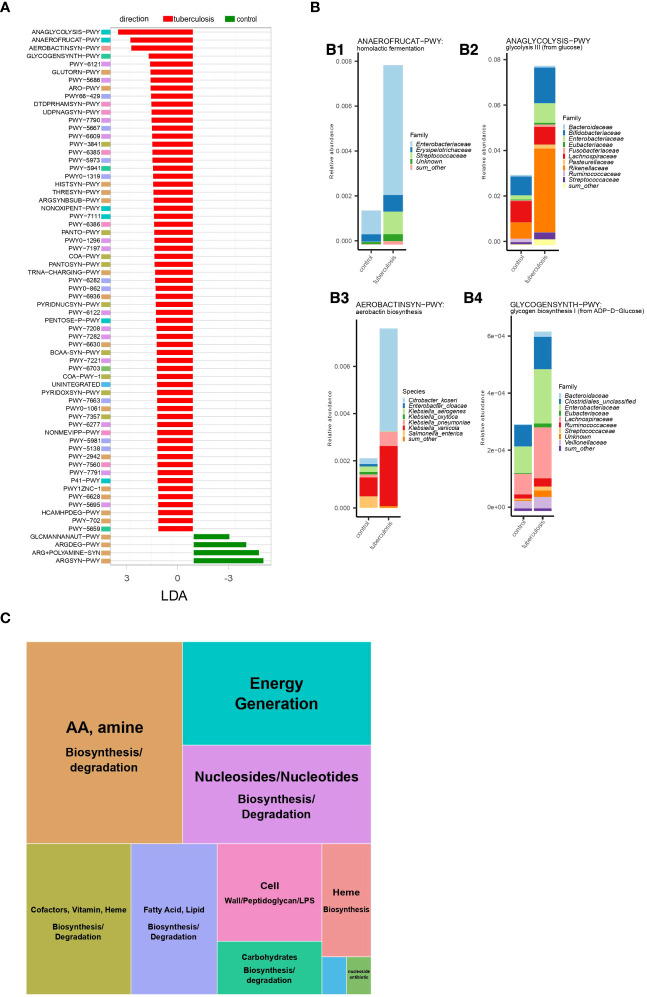
Microbial metabolic pathways increased in TB patients. **(A)**. Metabolic pathways were differentially enriched (LDA score > 2,5) in TB patients and healthy controls based on linear discriminant analysis. Metabolic pathway names represent MetaCyc identifiers. Full pathway names are given in **Supplementary Figure 3**. Colored boxes for each metabolic pathway indicate membership in the higher-level MetaCyc pathway types summarized in **Figure 3C**; **(B)**. Abundance of the four metabolic pathways enriched in TB patients and contribution of bacterial taxa; The total height of the stacked barplot corresponds to the fraction of the pathway from the total amount of pathways in the gut microbiome in TB patients. Each colored section of the stacked barplot represents different bacterial families’ contributions to this pathway. Thus, colored sections in B1 show the contribution of bacterial families to homolactic fermentation, in B2 to glycolysis III (from glucose), in B4 to glycogen biosynthesis I (from ADP−D−Glucose); in B3, sections represent bacterial species contributions to the aerobactin biosynthesis pathway; **(C)**. Summary of metabolic pathway types enriched in TB patients. The block size reflects the number of unique metabolic pathways belonging to a particular type of metabolic pathway.

The microbial community constantly adapts to the intestinal environment by increasing or decreasing certain species and, hence, certain gene assemblages and metabolic pathways. We inferred metabolic pathways via gene assemblages in the analyzed microbiomes using the HUMAnN 3.6 tool (Beghini et al., 2021). This approach allowed us to delineate quantitative changes in the metabolic potential of the gut microbes in patients. It is important to note that inferred metabolic potential/capacity does not imply activity. Rather, such inferences can reflect community response through an increase or decrease of the carriers of a pathway. In total, 131 metabolic pathways were differentially enriched between TB patients and controls’ gut microbiomes (adjusted p-value ≤ 0.05 and LDA-score ≥ 2). Notably, 120 pathways were overrepresented in TB patients’ microbiomes (p ≤ 0.05, **Supplementary Table 3**, **Supplementary Figure 4**), as compared to only 11 in HCs. When clustered into higher-level MetaCyc pathway groups, this increase in metabolic capacity revealed an elevation in biosynthetic processes supporting microbial cell division and growth (**Figures 4A, C**). This net increase in pathways supporting bacterial growth in patients’ gut contrasts with the metabolic profile in healthy donors, where metabolic pathways reflect the normal functioning of the bacterial cell (**Figure 4A**).

Microbial metabolism and growth in the anaerobic colon can be achieved by different strategies for obtaining energy and biosynthetic precursors. The choice of the strategy depends on the carbon source, its oxidation state, pH, and other factors in the intestine (Wolfe, 2005). Hence, dominant strategies can be informative about the changes in the colon environment linked to the pathology. We, therefore, highlighted major changes in the number of energy production pathways observed in the TB patients’ gut microbiome. Our analysis showed that the gut microbiome in TB patients is characterized by increased metabolic pathways aimed at glucose consumption and fermentation with end products such as acetate and lactate. Specifically, glycolysis (ANAGLYCOLYSIS-PWY), homolactic fermentation (ANAEROFRUCAT-PWY), pentose phosphate pathway (NONOXIPENT-PWY and PENTOSE-P-PWY), and pyruvate fermentation to acetate and (S)-lactate I (P41-PWY) were the top changes in TB patients gut microbiome (**Figures 4A, B1, 2**). If these pathways are active, higher use of glucose oxidation (glycolysis) compared to the HC microbiome can indicate higher glucose availability in patients’ intestines. Glycolysis supports a higher rate of generating ATP and biosynthetic precursors for dividing bacteria. A relatively higher presence of the pentose phosphate pathway also indicates higher glucose consumption and reactions in this pathway, which synthesize additional biosynthetic precursors needed for dividing cells (Folch et al., 2021). In this hypothesized scenario, the source of the higher glucose in the patient’s large intestine is unclear. Under homeostatic conditions, a small amount of simple sugars may reach the colon from the small intestine. In the large intestine, simple sugars arise from host- and diet-derived complex carbohydrates fermented mostly by *Bacteroidetes* and readily consumed by resident bacteria. In patients, however, we find depletion of *Bacteroidetes* and no increase in pathways degrading complex carbohydrates to explain the hypothesized higher amounts of simple sugars (**Supplementary Figure 4**, **Supplementary Table 3**).

A higher flux of glucose and increase in glycolysis in anaerobic conditions and pH in the normal range (5.5-7.5 in the large intestine lumen and 7.1-7.5 on the mucosal surface) (Nugent et al., 2001) usually leads to excretion of acetate (acetogenesis), ethanol, and format. However, our analysis suggests that the microbial community in patients has adapted by increasing members that ferment to lactate and acetate (P41-PWY, PWY-5100, P461-PWY, and ANAEROFRUCAT-PWY) compared to HCs gut microbiome (**Figures 4A, B1**; **Supplementary Table 3**). While acetate and lactate are consumed by host cells and cross-feeding bacteria, acetate is normally the most abundant among the colon’s short-chain fatty acids (SCFA) (Wolfe, 2005). In contrast, lactate is normally kept at very low concentrations by both host cells and lactic acid-degrading bacteria and has a higher potential to acidify the intestine (Louis et al., 2022). It is, therefore, notable that in TB patients’ gut microbiomes, lactic acid fermentation (homolactic fermentation) is among the top elevated pathways and may indicate a higher potential of the microbial community to cause lactic acidosis (**Figure 4B1**).

In line with our inference of higher bacterial growth in TB patients’ intestines, we observed increased biosynthetic pathways supporting cell wall synthesis (**Supplementary Figure 5**, **Supplementary Table 3**). For example, we identified an increase in pathways producing cell wall components such as peptidoglycan, lipopolysaccharide, and the enterobacterial common antigen (DTDPRHAMSYN-PWY; UDPNAGSYN-PWY; PWY-6386; PWY-7315; PWY0-1241; PWY-1269; PWY0-1586) (**Supplementary Figure 5**). For these pathways, we computed each bacterial genus and family’s contribution. This information allowed us to learn about bacterial taxa that are likely assembling cell walls at an increased pace (**Supplementary Figure 5**). Our analysis identified major contributors (taxa contributing more than 1% of the total) from the following families: *Enterobacteriaceae, Streptococcaceae, Desulfovibrionaceae, Lachnospiraceae, Ruminococcaceae, Eubacteriaceae, Clostridiales, Prevotellaceae, Selenomonadaceae, Hafniaceae, Bacteroidaceae*, and *Vibrionaceae*, belonging mainly to the phylum *Proteobacteria*, *Firmicutes*, and *Bacteroidetes*. Notably, several bacterial families, such as *Enterobacteriaceae, Streptococcaceae, Eubacteriaceae*, and others, that contributed to higher cell wall synthesis pathways also contributed to the higher presence of pathways for energy production through glycolysis and homolactic fermentation (**Figures 4B1-4**).

## Discussion

Case-control studies that use fecal samples and sequencing in clinical settings have limitations and strengths that we outline upfront to help with the interpretation. In clinical settings, upon admission, patients have only 1-2 days before they start anti-TB therapy. Hence, to characterize the gut microbiome before treatment, only a one-point fecal collection is realistic. Fecal-derived material is dominated by the microbial community of the large intestine, and we highlighted this nuance throughout the text. Furthermore, contrary to common perception, metagenomic sequencing informs only about changes in the proportion of microbes. It remains blind to changes in microbial load (Morton et al., 2019) unless measured with specialized methods (Galazzo et al., 2020). Despite these limitations common to many microbiome studies, the large effect size of tuberculosis on the microbiome (**Supplementary Figure 1**) allowed us to identify systematic changes in the relative abundance of gut microbes in TB patients with our small sample size. The large effect size of tuberculosis (Cohen’s d between 0.8 and 4.5 for different measurements) implies that the effect of diagnosis played a dominant role in shaping the observed alterations and overwhelmed the contributions of confounding factors, such as diet and lifestyle (Cohen’s d < 0.2) (Rahman et al., 2022). Nevertheless, in interpreting tuberculosis’s effect on the gut microbiome, one should take into account that active tuberculosis develops in genetically susceptible individuals (Abel et al., 2014) and, in addition, murine models suggest that TB infection itself can alter gut microbiome (Winglee et al., 2014). Hence, our inferences about TB patients’ microbiomes are likely to stem from the combined effect of infection and genetic background, which can’t be separated in our case-control study design. It is also worth reiterating that our inferences on microbial metabolic potential do not imply the activity of these pathways in gut microbes. Rather, observed shifts reflect the number of carrying bacteria as a long-term response to altered environments in the intestine. Except for sophisticated assembly-based approaches (Marcelino et al., 2023), current bioinformatic tools infer bacterial taxa and metabolic pathways separately, limiting our interpretation (Douglas and Langille, 2021). Nevertheless, we attempted to link metabolic pathways with specific taxa using marker genes (**Figure 4**). Finally, the cause-and-effect relationships of inferred alterations with the disease are unclear. Nevertheless, in the next section, we discuss possible causes and implications of the detected alterations for TB treatment by proposing working hypotheses.

Previous studies characterized taxonomic changes in TB patients’ gut microbiomes; however, how these changes influenced host immunity was unclear and required insight into microbial function. We used deep sequencing and showed that gut community alterations in TB patients are accompanied by increased metabolic pathways leading to cell division and growth. To interpret these metabolic shifts further, we paid special attention to pathways of energy acquisition, which, on the one hand, informs on the colon environment and, on the other hand, on the metabolic output of the community and, hence, on their potential impact on the host. We found that the patient’s intestine had an increased proportion of microbes with the enzymatic capacity to consume glucose via anaerobic glycolysis and to ferment, which hinted at an unhealthy colon environment compared to healthy donors (**Figures 3A, C**).

Higher use of glycolysis presumably indicates higher than usual glucose availability in the TB patient’s colon, which is a potent cue for most microbes to switch the metabolic program (Wolfe, 2015). The source of higher glucose in patients’ large intestines is unclear. Normally, in the large intestine, the majority of available carbohydrates that the host does not consume are diet- (i.e., fiber from plant foods) or host-derived (e.g., mucin, cellular debris) complex carbohydrates (polysaccharides); they are broken down by dominant residents of the colon, primarily *Bacteroides* and *Clostridiales* (Kamada et al., 2013). However, our taxonomic analysis showed that *Bacteroides* were depleted in TB patients. In contrast, *Proteobacteria (Enterobacteriaceae)*, such as *E. coli*, which cannot normally break down polysaccharides into simple carbohydrates, increased (**Figures 2B**, **4B1**). Not surprisingly, *Clostridiales*, which can consume complex and simple carbohydrates, were increased (**Supplementary Figure 3**).

Irrespective of the source, a higher flux of glucose causes most bacteria to prefer glycolysis to grow and divide rapidly (Wolfe, 2015). Indeed, glycolysis supports 110 times faster generation of biosynthetic precursors and energy in terms of ATP but rapidly produces lactate (Wolfe, 2015). Using species-specific marker genes, we identified that increased glycolysis (glycolysis III from glucose) in patients’ guts was explained by taxa that generally contain harmful species, except *Bifidobacteriaceae.* Namely, *Enterobacteriaceae* (*E. coli, Citrobacter freundii*, and *Klebsiella pneumoniae*), *Streptococcaceae (Streptococcus thermophilus, S. salivarius, S. parasanguinis, S. gallolyticus), Rikenellaceae* (*Alistipes dispar, A. communis*), and *Bifidobacteriaceae* (*Bifidobacterium longum, B. catenulatum, B. pseudocatenulatum*, and *B. breve*) (**Figure 4B2**).

Our findings show that homolactic acid fermentation is the next top shift in the gut microbiome metabolism in patients, which is consistent with the higher anaerobic glycolysis. Bacterial taxa featured in higher glycolysis were among the major contributors to increased capacity for homolactic fermentation. Namely, representatives of *Enterobacteriaceae* (e.g., *E. coli, C. freundii*, and *K. pneumoniae*) and *Streptococcaceae (e.g., Streptococcus thermophilus, S. salivarius, S. parasanguinis*, and *S. gallolyticus*), and *Erysipelotrichaceae (Clostridium innocuum*) (**Figure 4B1**). Elevated production of lactate and acetate is evidenced by an increase in other pathways producing these acids (**Figure 4A**). Our findings, namely increased capacity for lactic acid fermentation and major contributing taxa, are consistent with the experimental data showing that homolactic acid fermentation is used by lactic acid bacteria, such as *Lactobacillus, Lactococcus*, and many *Streptococci*, as well as members of the family *Enterobacteriaceae*. *Enterobacteriaceae*, in addition, can perform mixed-acid (heterolactic) fermentation.

The higher presence of pathways for lactic acid fermentation and glycolysis indicates a higher propensity of the gut community to acidify the intestine (Kamada et al., 2013; Wolfe, 2015). While the host tissue can absorb lactate, the gut community also responds by increasing the number of lactate-utilizing bacteria (Russell and Rychlik, 2001; Louis et al., 2022). We examined our taxonomic data to test this predicted compensatory increase in lactate-utilizing species. Our taxonomic data shows that several prominent lactate utilizers are strongly increased in TB patients at species or genus levels. Specifically, *Coprococcus catus* (Louis et al., 2022) and *Megasphaera BL7* (Shetty et al., 2013), which use the acrylate pathway, were strongly increased in TB patients (**Supplementary Table 2**). At the genus level, TB patients show enrichment of the two highly adapted lactate-utilizing genera, *Anaerobutyricum* and *Anaerostipes* (**Supplementary Figure 3**). While these observations are consistent with a compensatory increase in lactate utilizers, counting the abundance of lactate utilization pathways is not straightforward. Thus, the acrylate pathway is used only by a limited number of human gut bacteria, and most other lactate-utilization pathways involve conversion to pyruvate. Pyruvate makes lactate utilization intertwined with central metabolic pathways that can start with any major organic compound, i.e., carbohydrates, lipids, and amino acids. Nevertheless, the only testable acrylate pathway did not feature in patients according to linear discriminant analysis (**Figure 4A**) (Reichardt et al., 2014). We recognize that a more advanced approach might disentangle this complexity. However, our current methodology renders the accurate assessment of lactate utilization challenging, which is a limitation of current work.

The accumulation of lactate in the colon not only decreases pH and strongly inhibits the resident commensal flora from *Bacteroidetes* (Wang et al., 2020) but also provides a carbon source for competitors (e.g., some pathogenic species from *Proteobacteria* or lactate-utilizing species from Firmicutes) (Louis et al., 2022). Our taxonomic analysis showed a systematic decrease in *Bacteroidetes* proportion in patients and an increase in *Actinobacteria, Proteobacteria*, and *Firmicutes* (**Figure 2A**). Observed taxonomic changes closely mirrored the experimentally established effects of lactate acidosis on the human colon microbiota such as depletion of *Bacteroidetes* and increase of *Actinobacteria, Proteobacteria*, and some *Firmicutes* that are tolerant to low pH (Wang et al., 2020). This experiment showed that lactic acidosis destabilized resident flora and promoted pathogenic bacteria, such as *Streptococcus bovis, Salmonella, E. coli, Campylobacter jejuni, C. difficile*, and *Vibrio cholerae*, that are adapted to low pH and benefit from lactate consumption (Louis et al., 2022). Our taxonomic analysis also showed an increased abundance of similar or closely related pathobionts in TB patients, such as *E. coli, C. freundii*, *K. pneumoniae*, *C. innocuum*, and different species in *Streptococcaceae*, that are associated with diverse human diseases (Crum-Cianflone, 2009; Chhatwal and Graham, 2017; Santos et al., 2020; Li et al., 2022; Xue et al., 2023). It should be noted that observed taxonomic changes broadly resemble the effect of mycobacterium infection reported in murine models of TB infection (Winglee et al., 2014). Hence, observed taxonomic changes and altered metabolic capacity might be sequelae of the altered host physiology - hypoxia in the host tissues, the overall increase in glycolysis, and lactate secretion during immune response in TB (Llibre et al., 2021). Irrespective of the cause, taxonomic changes alone are profound - the combined relative abundance of gut pathobionts in patients reaches 1/4 of the total microbiome content (**Figures 3A, B**). The extent of the discovered pathobiont burden likely poses a heavy toll on host immunity on top of the mycobacterium infection.

Since our metabolic reconstructions suggest lactate acidosis, we conclude by discussing lactate’s direct effects on the course of MTB infection and TB treatment outcome. In clinical tuberculosis, the balance between the antibacterial activity of macrophages and tissue destruction by extracellular enzymes, primarily matrix metalloproteinases (MMPs), produced during inflammation is critical to disease outcome and transmission (Ong et al., 2014). Recent studies show that this balance is affected by the extracellular concentration of lactate (Whittington et al., 2023), produced by infected macrophages using glycolysis (Langston et al., 2017). Mechanistically, lactate, on the one hand, improves clearance of MTB in the already infected macrophage by promoting autophagy. On the other hand, it markedly suppresses both TNF-α and IFN-γ but leaves IL6 and IL10 unaffected, thereby interfering with further enhancement of anti-MTB activity (Maoldomhnaigh et al., 2021). Moreover, lactate specifically upregulates matrix metalloproteinases that cause lung destruction in the tuberculosis (Whittington et al., 2023), the process known to result in higher morbidity and mortality in the TB treatment (Ong et al., 2014). Thus, recent mechanistic insights underscore the multifaceted effect of lactate on Th1 response with an overall negative impact on the ability to cope with MTB, which is more in line with clinical data. Indeed, serum lactic acidosis is associated with higher mortality during TB treatment (Ntambwe and Maryet, 2012; Im et al., 2021) and other infections (Trzeciak et al., 2007), and monitoring lactate acidosis was shown to help rescue TB patients (Im et al., 2021).

In the context of ongoing inflammation, high lactate in patients’ serum can also be attributed to the glycolytic activity of immune cells and an inflamed tissue environment (Manosalva et al., 2021). Our findings point to an additional unacknowledged source for high lactate in the patients’ sera, the gut microbiota. Thus, in light of lactate’s importance in disease outcome, our findings point to an intriguing avenue to understand the role of gut microbiota in TB treatment efficacy. Namely, collecting clinical data along with serum and gut microbiome readouts, such as fecal metabolites and deeply sequenced metagenomes, to directly assess sources of intestinal lactate acidosis, it’s possible impact on serum levels, and TB outcome. Importantly, the idea to target lactic acidosis in TB treatment as a new host-directed therapy was proposed earlier by others (Kiran and Basaraba, 2021). In animal studies, excess fermentation in the gut has long been known to cause lactic acidosis, and treatment measures are actively studied (Nagaraja and Titgemeyer, 2007). Here, we add that one major source of lactic acidosis can be intestinal microbiota-driven acidosis.

## Data availability statement

The shotgun metagenomic data used in this study are available in the NCBI database under BioProject accession code PRJNA1061168. The data is available for research purposes upon request. Requests should be directed to the corresponding author Bayazit Yunusbayev (yunusbb@gmail.com).

## Ethics statement

The studies involving humans were approved by the Ethics Committee of the Republican Clinical Antituberculous Dispensary (Russia). The studies were conducted in accordance with the local legislation and institutional requirements. The participants provided their written informed consent to participate in this study. Written informed consent was obtained from the individual(s) for the publication of any potentially identifiable images or data included in this article.

## Author contributions

MY: Funding acquisition, Methodology, Project administration, Supervision, Visualization, Writing – original draft, Writing – review & editing, Conceptualization, Investigation. LB: Conceptualization, Investigation, Methodology, Project administration, Writing – original draft. DT: Formal analysis, Methodology, Software, Validation, Writing – original draft. AB: Formal analysis, Methodology, Software, Validation, Writing – original draft. AZ: Investigation, Methodology, Validation, Writing – original draft. SB: Investigation, Methodology, Project administration, Validation, Writing – original draft. RA: Formal analysis, Software, Validation, Visualization, Writing – original draft. FB: Data curation, Formal analysis, Investigation, Methodology, Supervision, Validation, Visualization, Writing – review & editing. BY: Conceptualization, Data curation, Project administration, Resources, Software, Supervision, Visualization, Writing – original draft, Writing – review & editing.
