# Supplementary material for: Excess fermentation and lactic acidosis as detrimental functions of the gut microbes in treatment-naive TB patients
Source: Front Cell Infect Microbiol. 2024 Feb 19;14:1331521. doi: 10.3389/fcimb.2024.1331521 (PMC10910113; doi:10.3389/fcimb.2024.1331521)
Supplement: Supplementary file 1 [file DataSheet_1.docx]

**Supplementary Information**

Table of Contents

Supplementary Table S1. Clinical and demographic characteristics of the compared groups 2

Supplementary Figure S1. Effect size estimates and power analysis 3

Supplementary Figure S2. Taxonomic composition of the fecal microbiota of TB patients and healthy controls 4

Supplementary Figure S3. Taxonomic features of the gut microbiota of TB patients and controls 5

Supplementary Figure S4. Metabolic features of the gut microbiota of TB patients and controls 6

Supplementary Figure S5. Metabolic pathways of cell wall biosynthesis of the gut microbiota of TB patients

and controls 7

**Supplementary Table S1. Clinical and demographic characteristics of the compared groups**

|  | **TB patients**  **(N =23)** | **Healthy control**  **(N =47)** |
| --- | --- | --- |
| Female | 9/23 (39%) | 22/47 (47%) |
| Men | 14/23 (61%) | 25/47 (53%) |
| Age, years (Median (IQR) | 44 (38-53) | 40 (36-45) |
| Clinical forms of pulmonary tuberculosis | |  |
| Infiltrative TB | 15/23 (65,2%) | .. |
| Disseminated TB | 7/23 (30,4%) | .. |
| Caseous pneumonia | 1/23 (4,3%) | .. |

Data are median (IQR) (first and third quartiles) or n/N (%), unless stated otherwise. N ― number of individuals


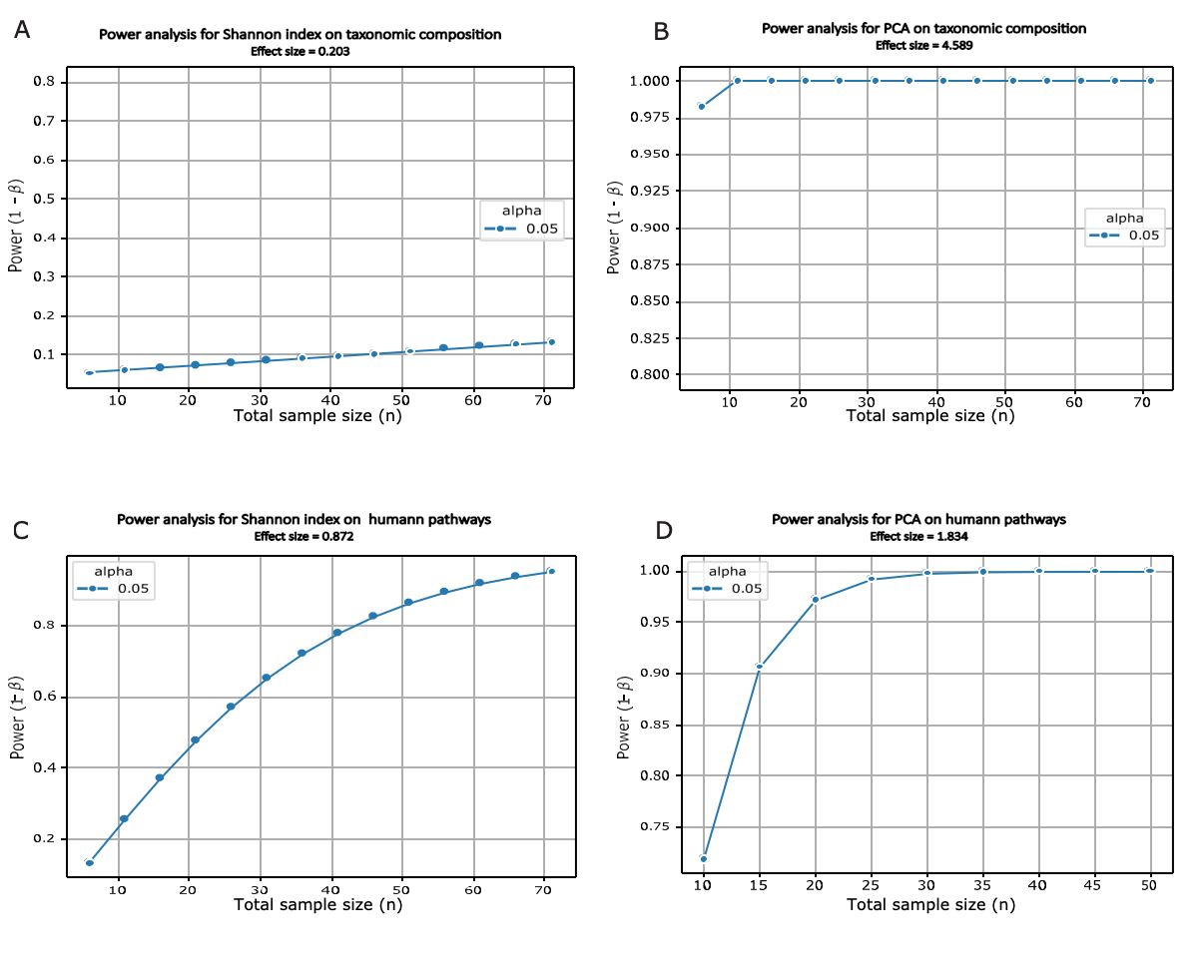
**Supplementary Figure S1. Power analysis of tuberculosis as a binary category.**

Power curves were computed at a significance level of 0.05. (A) Power estimates given the effect size of tuberculosis (Cohen's d=0.203) on taxonomic diversity (Shannon index) (B) Power estimates given the effect size (Cohen's d=4.589) of tuberculosis on taxonomic variation captured by the first principal component (PC1), (C) Power estimates given the effect size of tuberculosis (Cohen's d=0.872) on metabolic pathway diversity (Shannon index) as inferred using Humann analysis, (D) Power estimates given the effect size of tuberculosis (Cohen's d=1.834) on metabolic pathway variation captured by the first principal component (PC1).


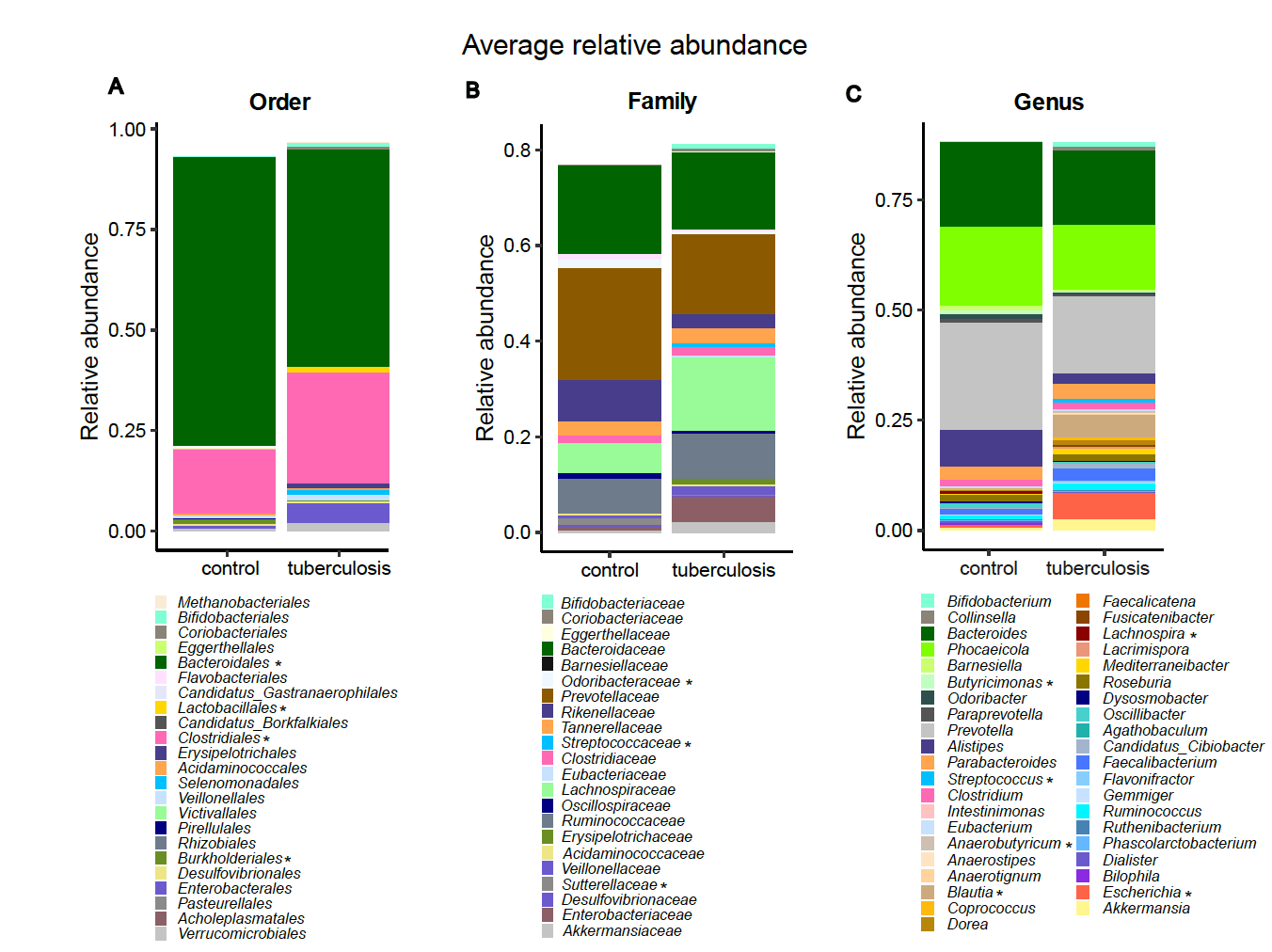


**Supplementary Figure S2. Taxonomic composition of the fecal microbiota of TB patients and healthy controls.**

Plots show the average relative abundance of microbial taxa in the gut microbiota of TB patients and controls at the order (A), family (B), and genus (C) levels. A statistical difference in proportions between groups was tested using the ANCOM statistical framework. An asterisk indicates taxa that are statistically significantly different between the compared groups (P < 0,05). We required bacterial taxa to be present in 60% of donors at an abundance of 5% or more. In addition, we removed unclassified taxa (such as FGB, GGB, etc.) for the visualization. Taxonomic composition at the genus, family, and order levels is strongly altered in TB patients.


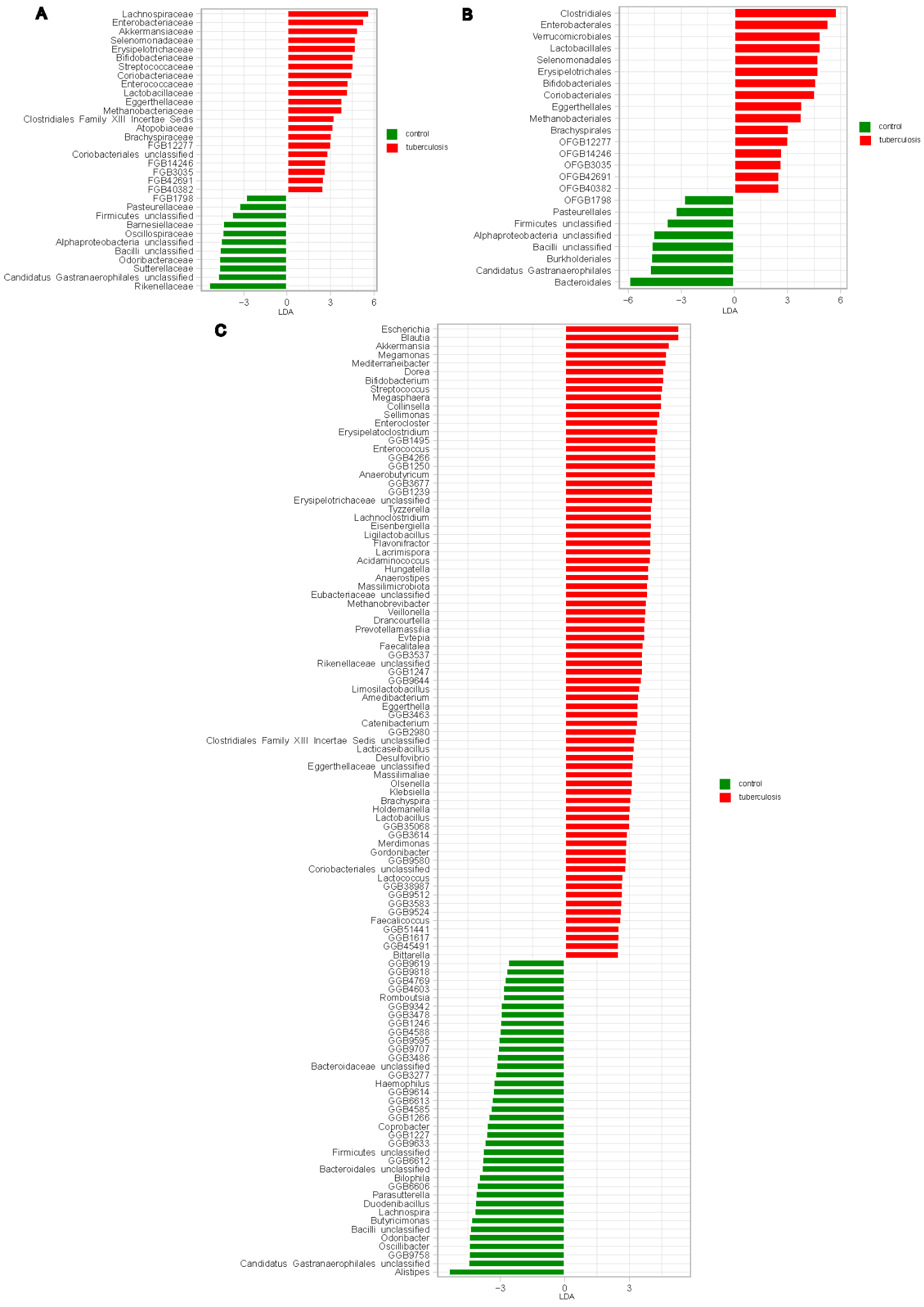


**Supplementary Figure S3. Taxonomic features of the gut microbiota of TB patients and controls.**

LDA score plots were generated using the LEfSe analysis. The length of the bar column represents the LDA score. Plots show the microbial taxa with significant differences between the TB patients (red) and healthy controls (green) at the order (A), family (B), and genus (C) level (LDA score > 2,5). Two prominent lactate utilizers that use the acrylate pathway, *Coprococcus catus* and *Megasphaera BL,* were strongly increased in TB patients. Family *Clostridiales*, which can consume both complex and simple carbohydrates, were increased in TB patients.

.

**
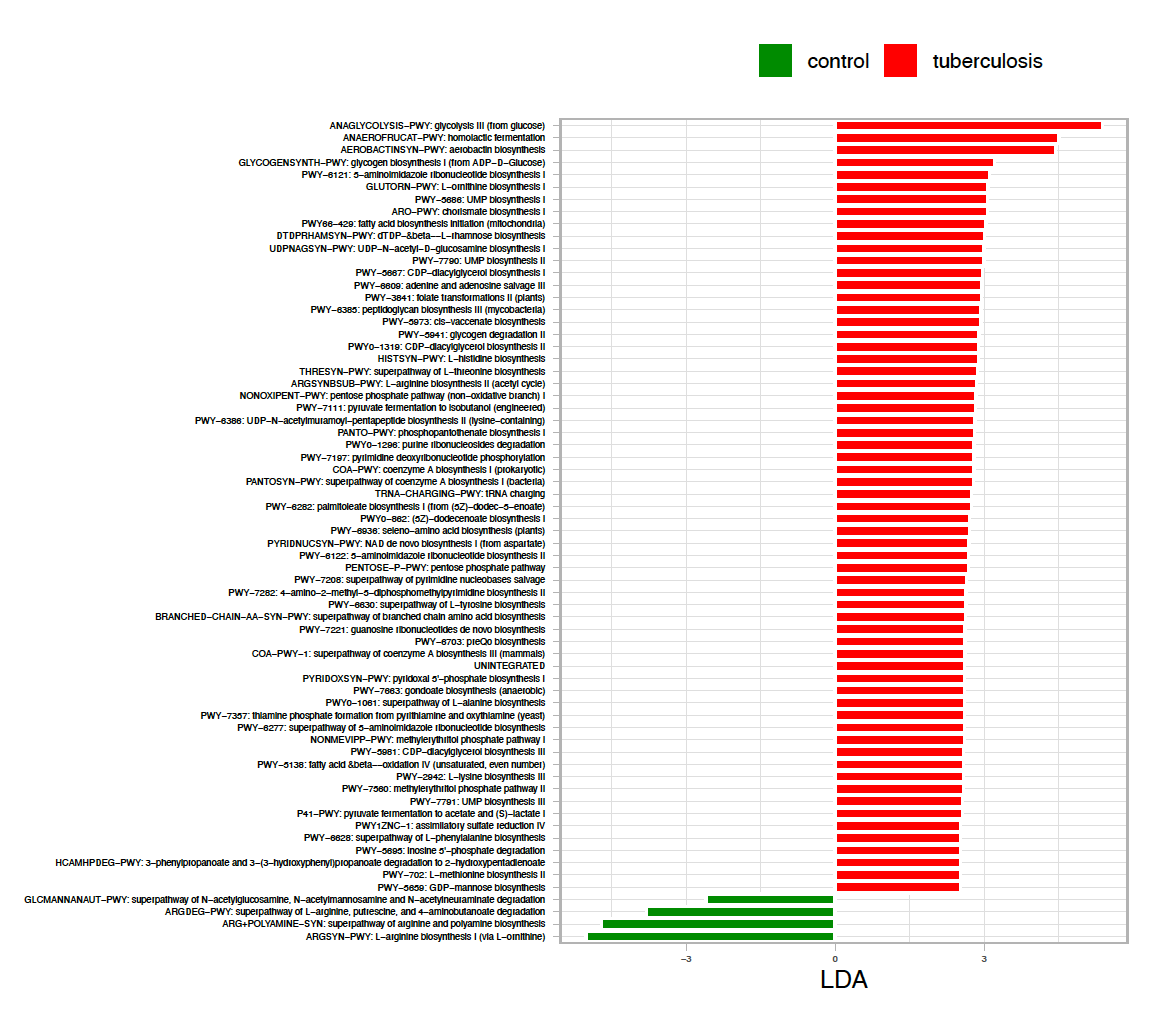
**

**Supplementary Figure S4. Metabolic features of the gut microbiota of TB patients and controls.**

LDA score plots were generated using the LEfSe analysis. The length of the bar column represents the LDA score. Plots show metabolic pathways with significant differences between the TB patients (red) and healthy controls (green) (LDA score > 2,5). Metabolic pathways are inferred based on HUMAnN 3.6 and annotated based on Metacyc. In the gut microbiota of TB patients, there is an increase in the number of pathways supporting bacterial growth, while the metabolic profile of healthy donors reflects normal bacterial cell function.


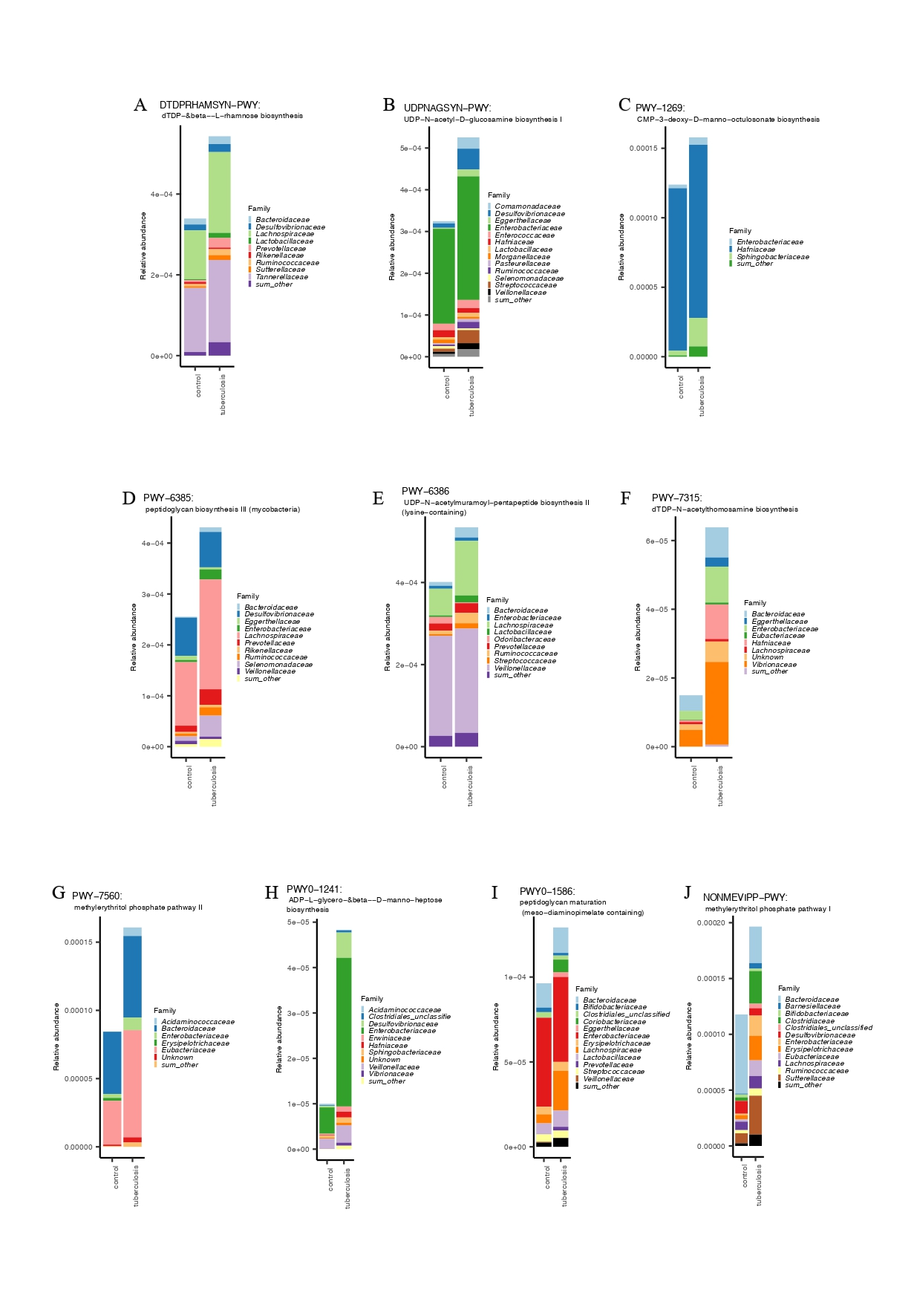


**Supplementary Figure S5. Metabolic pathways of cell wall biosynthesis of the gut microbiota of TB patients and controls.**

A - Relative abundance of dTDP-&beta--L-rhamnose biosynthesis pathway in the gut microbiota of TB patients and controls; B - Relative abundance of UDP-N-acetylmuramoyl-pentapeptide biosynthesis II (lysine-containing) pathway in the gut microbiota of TB patients and controls; C - Relative abundance of CMP-3-deoxy-D-manno-octulosonate biosynthesis pathway in the gut microbiota of TB patients and controls; D - Relative abundance of peptidoglycan biosynthesis III (mycobacteria) pathway in the gut microbiota of TB patients and controls; E - Relative abundance of UDP-N-acetylmuramoyl-pentapeptide biosynthesis II (lysine-containing) pathway in the gut microbiota of TB patients and controls; F - Relative abundance of dTDP-N-acetylthomosamine biosynthesis pathway in the gut microbiota of TB patients and controls; G - Relative abundance of methylerythritol phosphate pathway II in the gut microbiota of TB patients and controls; H - Relative abundance of ADP-L-glycero-&beta--D-manno-heptose biosynthesis pathway in the gut microbiota of TB patients and controls; I -Relative abundance of peptidoglycan maturation (meso-diaminopimelate containing) pathway in the gut microbiota of TB patients and controls; J - Relative abundance of methylerythritol phosphate pathway I in the gut microbiota of TB patients and controls. The plots show the relative abundance of metabolic pathways in TB patients and healthy donors and the contribution of bacterial taxa. Metabolic pathways are inferred based on HUMAnN 3.6 and annotated based on MetaCyc. The TB patient group has a higher abundance of metabolic pathways related to cell wall synthesis than the control group.
